# A combined experimental and theoretical study of the prototypical polymorphic transformation from marcasite to pyrite FeS_2_†

**DOI:** 10.1039/d4dt03447c

**Published:** 2025-02-19

**Authors:** KeYuan Ma, Ulrich Aschauer, Fabian O. von Rohr

**Affiliations:** a Max Planck Institute for Chemical Physics of Solids 01187 Dresden Germany; b Department of Chemistry and Physics of Materials, University of Salzburg 5020 Salzburg Austria; c Department of Quantum Matter Physics, University of Geneva CH-1211 Geneva Switzerland Fabian.vonRohr@unige.ch

## Abstract

We present an investigation of the prototypical polymorphic structural transformation from marcasite to pyrite FeS_2_ studied by combining annealing experiments and theoretical calculations. These experiments have become possible due to the availability of laboratory-synthesized high-purity marcasite samples. We constructed an annealing temperature, time, and phase composition map of marcasite based on a series of isothermal annealing experiments at different temperatures and heating times. To understand the microscopic mechanisms and pathways of the transformation, we performed theoretical calculations that yield an agreement with the experimental results. Based on the combined results, we show that the transformation of marcasite to pyrite, while thermodynamically favorable, is hindered by a kinetic barrier of the order of 3 eV. As a result, marcasite can remain stable for extended times at temperatures below 450 °C.

## Abstract 2

TOC illustrates the polymorphic transformation of FeS_2_ from marcasite to pyrite, highlighting the energy barrier (Δ*G*) associated with the phase transition.

## Graphical abstract

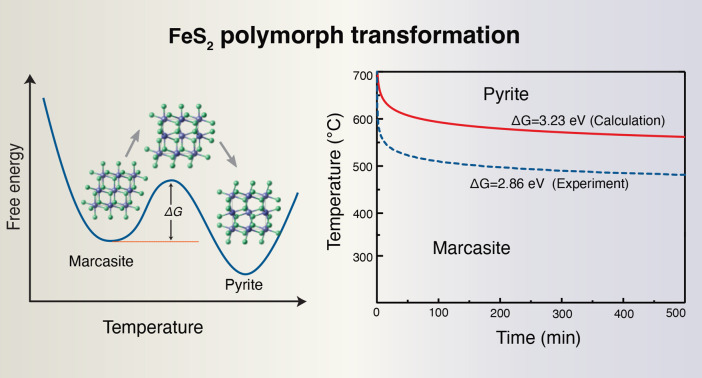

## 1 Introduction

A polymorphic phase transformation is a change in the crystal structure of a material without changing the chemical composition.^1,2^ These transitions can significantly affect the material's physical properties. Iron disulfide (FeS_2_) has two naturally occurring polymorphs, namely marcasite and pyrite, with distinct differences in crystal structures and physical properties:^3^ marcasite crystallizes in an orthorhombic structure, while pyrite has a cubic structure, both structures are made of iron sulfide octahedra. Marcasite is a metastable phase that can be irreversibly converted to pyrite when subjected to high temperatures or pressure.^4^ Recently, lab-synthesized high-purity samples of marcasite FeS_2_ have become available through acidity-controlled hydrothermal reactions.^5^

The marcasite-to-pyrite transformation is an irreversible, solid-to-solid phase transition that takes place without any intermediate liquid phases.^6^ This transition is widely regarded as a prototypical polymorphic phase transformation, as both polymorphs are naturally occurring and chemically comparably simple. Furthermore, FeS_2_ polymorphs, especially pyrite, consisting solely of non-toxic, and earth-abundant elements, are widely considered promising candidates for various electronic and catalytic applications.^7–9^ Understanding the mechanisms of the conversion from marcasite to pyrite has important implications for understanding the preparative chemistry of FeS_2_, and the applications of FeS_2_ in various areas.

Especially, mineralogists have investigated this transformation process with various techniques, including *in situ* heating X-ray diffraction and infrared spectroscopy approaches. These approaches have provided averaged structural information from millions of naturally occurring microcrystals.^4,10,11^ These studies provide some insights into the structural aspect and kinetics of the transformation.^4,11^ However, a comprehensive mechanistic and kinetic study of the transformation was difficult due to the complexity of factors that influence the process such as temperature, particle size, impurities, and defects. This is especially difficult for marcasite samples from a natural source, as some inborn impurities and defects are inevitable and challenging to qualify and quantify.^11^ These undesired factors can affect the measured results in many unknown aspects, leading to discrepant results between different works.

Apart from those experiments, the stability of FeS_2_ polymorphs has also been investigated by theoretical calculations.^12–17^ It was shown that the employed density functional plays a crucial role in determining the pyrite-marcasite phase stability.^12,13,17^ Based on total-energy calculations at finite pressure, it was found that marcasite can convert to pyrite at pressures above 2.8 GPa to 9.0 GPa, the exact transition pressure being functional dependent.^13,14,16^ At even higher pressures, marcasite is predicted to convert to other phases.^15^ However, these calculations performed under high pressures lack experimental support, and they cannot explain the marcasite-to-pyrite transformation occurring at high temperatures. While comparative studies of the two phases exist,^12,13,16^ until now, no calculations of this polymorphic transformation exist that would provide a deeper atomic-level understanding of the underlying mechanisms and pathways.

Here, we carried out studies to investigate the marcasite to pyrite phase transformation utilizing a combination of thermal annealing experiments on lab-synthesized high-quality marcasite crystals and theoretical calculations. By integrating experimental observations with theoretical calculations, we aim to gain atomic-level insights that will rationalize the experimental observations and transition kinetics of this phase transformation. These results provide new atomic-level insights into the marcasite-to-pyrite polymorphic phase transformation mechanism and kinetics.

## 2 Experimental section

### 2.1 Growth of marcasite crystals

The marcasite crystals used in this study were prepared by a space-separated hydrothermal process according to our previous work.^5^ A steel autoclave with a large PTFE liner with a volume of 115 ml was filled with 30 ml of a 1 M sodium thiosulphate solution (Na_2_S_2_O_3_·5H_2_O, ≥99.5%). Then, a smaller PTFE beaker with a volume of 15 ml containing 10 ml of a 1 M FeCl_2_·4H_2_O (99.99%) solution, was placed inside the middle of the large PTFE liner. The autoclave was closed and heated at 240 °C for 2 days in a furnace. After the reaction, it was cooled to room temperature by removing it from the furnace. Marcasite crystals were formed above the surface of FeCl_2_ solutions and coated on the walls of the PTFE beaker. The collected crystals were washed sequentially with distilled water, toluene, and ethanol to allow maximum dissolution of sulfur. Finally, the crystals were dried under N_2_ gas at room temperature for further characterization.

### 2.2 Thermogravimetry analysis of lab-prepared marcasite

A thermogravimetry differential thermal analysis (TG-DTA) measurement of lab-prepared marcasite was performed on a Netzsch Jupiter STA 449 F3 TGA in a pure Ar atmosphere. 30 mg of marcasite were placed in a small Al_2_O_3_ crucible at a temperature range of room temperature to 800 °C at a heating rate of 2 °C min^−1^. After measurement, the residual products were collected for powder X-ray diffraction measurement.

### 2.3 Isothermal annealing experiments of lab-synthesized marcasite

We conducted a series of isothermal annealing experiments at temperatures between *T* = 400 and 700 °C with different heating times under argon atmosphere. Large lab-prepared marcasite crystals were crushed in an agate mortar to fine powders (particle size ≤40 μm). 30 mg marcasite powders were sealed into a series of evacuated quartz tubes under 1/3 atm argon. The temperature of a box furnace was set to a certain predefined value. When the temperature became stable, 10–20 evacuated quartz tubes filled with 30 mg marcasite powder each were quickly put into the middle of the furnace and the annealing time started. After annealing for a determined amount of time, the tubes were quenched in cooled water. After well over one hundred such cycles, a series of quenched marcasite samples with different annealing times and temperatures were obtained. All these samples were prepared for powder X-ray diffraction (PXRD) measurement to analyze the final phase and composition.

### 2.4 Diffraction

The phase purity and crystal structure measurements of the samples were performed by powder X-ray diffraction (PXRD) measurements in transmission mode on an STOE STADIP diffractometer with Mo Kα radiation (*λ* = 0.7093 Å). The PXRD patterns were collected in the 2*θ* range of 10–40° and a scan rate of 0.25° min^−1^.

### 2.5 Computational method

Our density functional theory (DFT) calculations were performed with the VASP code.^20–23^ The computational setup and in particular the employed density functional is known to have a notable effect on the energy balance of the pyrite and marcasite phases and the equilibrium volume, while other properties such as the electronic structure are not largely affected by the functional.^12^ At odds with experiment,^19^ PBE as well as GGA+U functionals, predict marcasite to be more stable than pyrite.^16^ More recent GGA functionals such as PBEsol and AM05, on the other hand, yield the correct stability of the phases,^12^ while, however, still underestimating the volume, which is most closely reproduced by PBE.^13^ Somewhat remarkably, LDA also yields the correct phase stability, while, as usual, underestimating the volume.^13^ In our test calculations, we were unable to reproduce the superior performance of AM05 compared to LDA, finding (in agreement with ref. 13) very similar energy differences between the two phases but (unexpectedly) also very similar volumes as shown in Table 1. Since our test calculations also yielded similar transition state energies for a trial SSNEB pathway (4.35 eV with LDA and 4.44 eV with AM05 at their respective relaxed volumes) and hence a similar description of the bond reconfiguration, we decided to adopt the more established LDA density functional for our calculations.

**Table 1:** Comparision of computed and experimental properties

Phase	Property	LDA	AM05	Expt.
Pyrite	Volume (Å^3^ u.c.^−1^)	146.806	148.243	158.9^18^
	*a* = *b* = *c* (Å)	5.275	5.292	5.416^18^
Marcasite	Volume (Å^3^ u.c.^−1^)	75.765	76.561	81.64^19^
	*a* (Å)	4.325	4.337	4.444^19^
	*b* (Å)	5.286	5.303	5.425^19^
	*c* (Å)	3.314	3.328	3.386^19^
	Rel. energy (meV f.u.^−1^)	7.8	9.2	43.4^19^

We employed projector augmented wave (PAW) potentials^24,25^ with Fe(3s, 3p, 3d, 4s) and S(3s, 3p) valence electrons together with a cutoff of 500 eV for the kinetic energy of the plane-wave basis. Calculations were performed spin-polarized with an initial ferromagnetic spin arrangement of the Fe ions but converged to non-magnetic Fe^2+^ octahedral low-spin d^6^ solutions for both phases. Structures were relaxed until forces converged below 10^−3^ eV Å^−1^ and stresses below 5 × 10^−5^ eV Å^−3^.

For our solid-state nudged elastic band (SSNEB)^26,27^ calculations, we employed a supercell of marcasite that has lattice vectors 
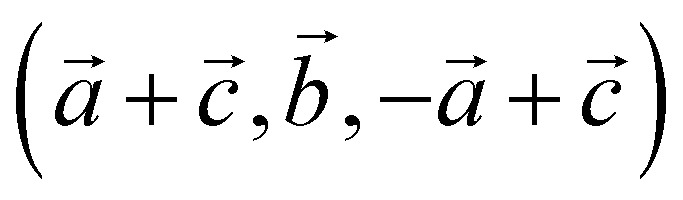
 with respect to the marcasite unit cell. This cell has the same number of formula units and similar Fe positions as the pyrite cell but one cell angle different from 90° (74.55°). Reciprocal space for this cell was sampled with 4 × 4 × 4 Monkhorst–Pack^28^ meshes. Climbing-image SSNEB calculations were performed with 7 images. Phonon calculations to test for dynamical instabilities and to compute phonon frequencies for the partition functions were performed with phonopy^29^ using symmetry inequivalent displacements of ±0.01 Å.

## 3 Results and discussion

### 3.1 Overview of marcasite and pyrite structure relevance

**Fig. 1:**
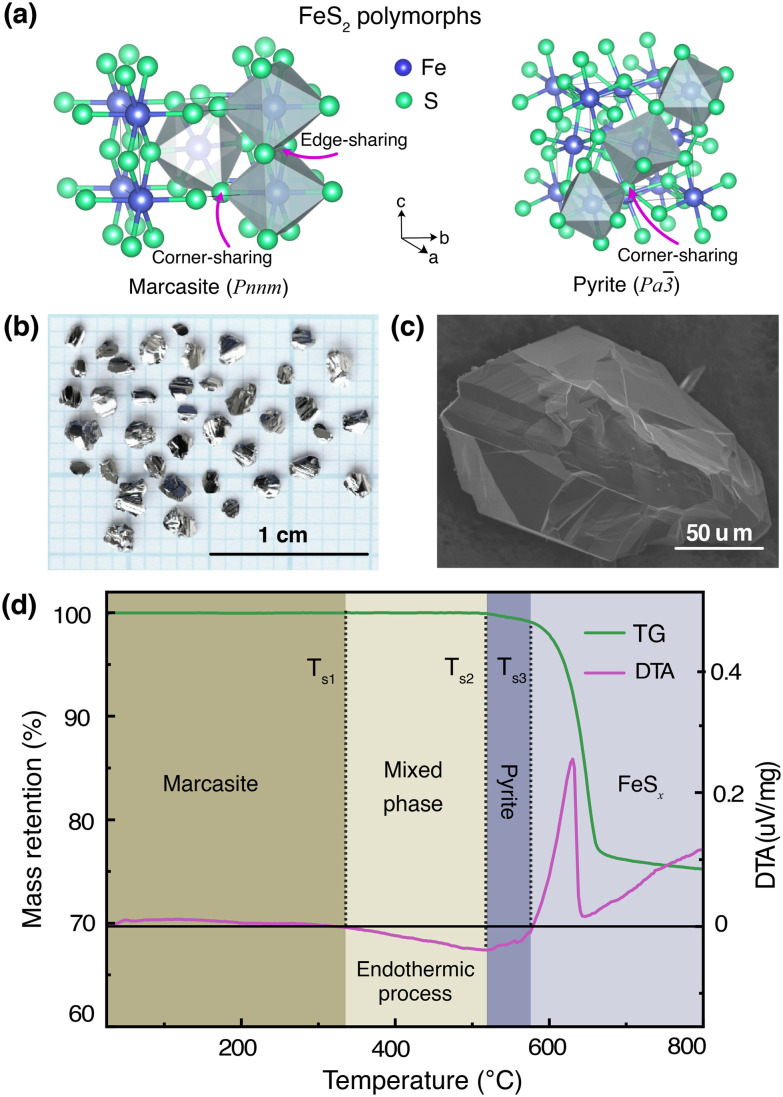
(a) Crystal structures of the FeS_2_ polymorphs, (b) photograph of marcasite single crystals obtained from a space-separated hydrothermal synthesis, (c) SEM image of a marcasite single crystal (d) TG and DTA measurements of marcasite from room temperature to 800 °C at a heating rate of 2 °C min^−1^ under argon atmosphere.

In Fig. 1(a), we show the crystal structures of the FeS_2_ polymorphs – marcasite and pyrite, respectively. The structures of these two polymorphs display differences, but also certain similarities. Both marcasite and pyrite structures have FeS_6_ octahedra as their elemental building blocks, where the Fe^2+^ cations are octahedrally coordinated by covalently bonded disulfide S_2_^2−^ ions with short bonding distances. The distinct structure difference of marcasite and pyrite lies in the connections of the FeS_6_ octahedra. In marcasite, the octahedral FeS_6_ units are edge-shared along the unit cell *c*-axis and corner-linked in *a*, *b*-axis directions. Whereas in pyrite, all the FeS_6_ octahedra are corner-linked with Fe situated at the face-centered cubic sites. The differences in FeS_6_ octahedron connectivity between marcasite and pyrite impact their properties, with pyrite's corner-sharing structure providing higher density, mechanical hardness, and thermodynamic stability, while marcasite's edge-sharing connectivity creates anisotropy, lower stability, and susceptibility to degradation.^30,31^ This structural distinction also influences phase transformation, as marcasite, being metastable, can convert to pyrite over time through bond rearrangement to reduce internal strain and achieve a lower-energy configuration.^30–32^ Interestingly, it can be noted that the marcasite {101} and pyrite {001} planes show nearly the same atomic arrangement, and the Fe–Fe atomic distance of marcasite is very similar to that of pyrite with a minimal mismatch.^3,11,33^

To mitigate the undesired effects introduced by impurities and structural defects – commonly found uncontrollably in naturally occurring marcasite crystals – we employed a space-separated hydrothermal synthesis technique. This method enabled us to produce phase-pure marcasite single crystals, which were subsequently utilized in the present phase transformation studies. In Fig. 1(b), we present a digital photograph of single crystals of marcasite as obtained by employing the described hydrothermal synthesis method. In Fig. 1(c), we show an SEM image of a selected marcasite single crystal. All obtained crystals have a silvery, shiny metallic luster, and some of them have perfect exposed crystal surfaces. Unlike natural marcasite mineral samples, which usually have some inborn impurities and defects, our marcasite single crystals are phase pure without sulfur deficiencies, displaying a clear diamagnetic response, as expected in the absence of magnetic iron impurities.^5^

### 3.2 TG-DTA analysis

We have investigated the thermal stability of marcasite by a TG-DTA measurement from room temperature to 800 °C under an argon atmosphere, as shown in Fig. 1(d). At temperatures below 340 °C, there are no obvious changes in the TG and DTA curves of marcasite, indicating the compound is stable in this temperature range. Subsequently, the DTA profile reveals a very broad endothermic peak in a large temperature range from 340 to 525 °C without weight loss in the TG curve. This endothermic process without weight loss can be well attributed to the known irreversible marcasite to pyrite phase transformation, indicating marcasite needs to absorb external energy to overcome the transition energy barrier to nucleation of the pyrite phase. Thereafter, we observed a strong narrow exothermic peak between 585 °C and 625 °C in the DTA profile, accompanied by a sharp rapid weight loss in the TG curve. This process can be attributed to the pyrite decomposition process to iron(ii) sulfide:^34^FeS_2_ = FeS_*x*_ + (2 − *x*)S.

The final broad exothermic line tail above 650 °C can be assigned to the crystallization process of FeS_*x*_, which releases energy. The residual product of the TG-DTA measurement was checked by a PXRD measurement, and identified to be phase-pure FeS_*x*_ in the NiAs-type structure (S-Fig. 1, ESI†).

### 3.3 Chemical state of Fe and S in marcasite and pyrite

**Fig. 2:**
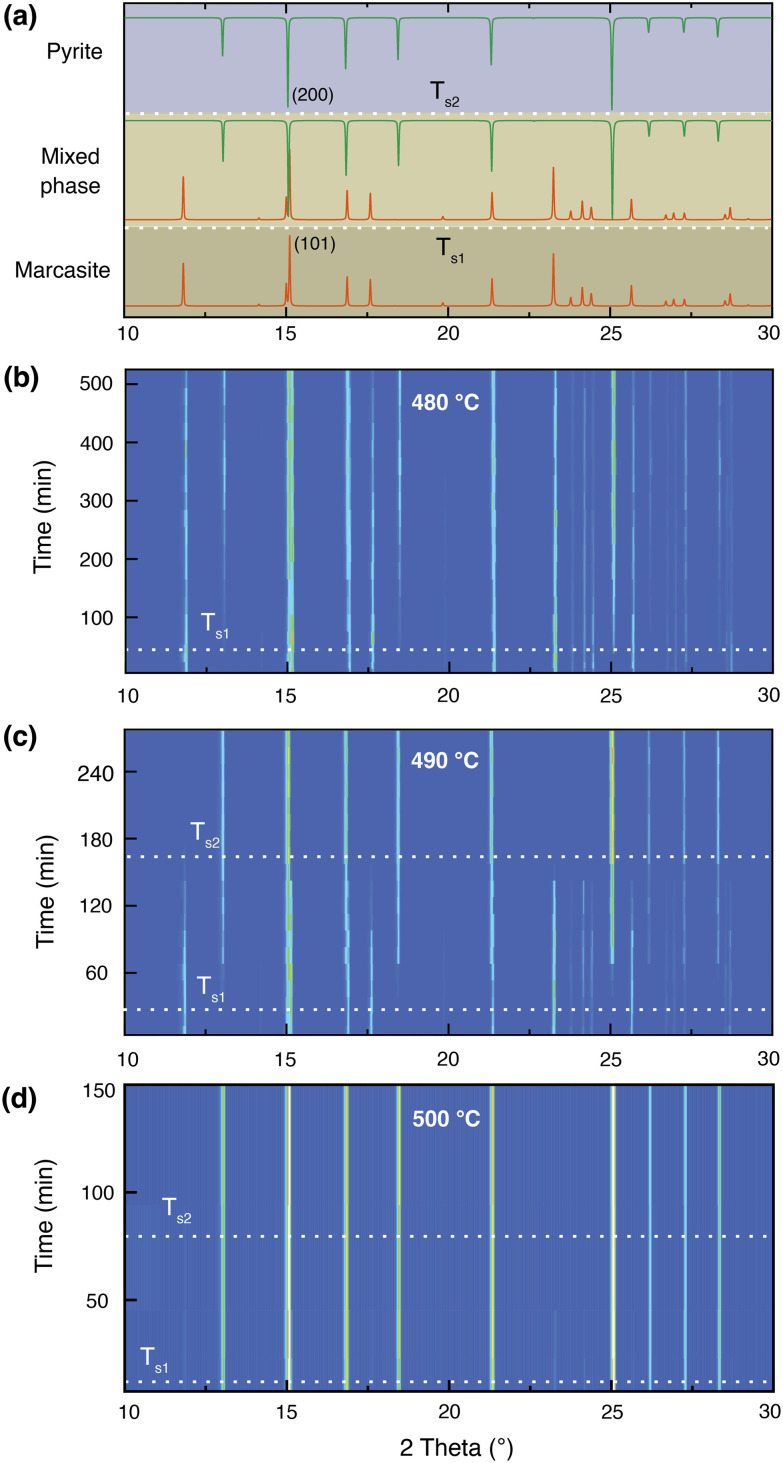
(a) Standard PXRD patterns of marcasite, mixed phase, and pyrite for reference. Isothermal annealing experiments of lab-prepared marcasite at different temperatures and times: (b) 480 °C, (c) 490 °C, and (d) 500 °C. For clear observations, the diffraction peaks in the 2*θ* range of 30–40° are not shown.

The different arrangements of the iron and sulfur atoms in the unit cells result in different shapes and symmetries of the crystals. Here, we performed X-ray photoelectron spectroscopy (XPS) measurements on marcasite and pyrite to analyze the chemical state of Fe and S in the two structures, as shown in S-Fig. 2(a) & (b) (ESI†). The Fe 2p spectra of marcasite and pyrite show no obvious difference in profile shapes, with two strong peaks coming from Fe 2p3/2 and Fe 2p1/2 spin orbits, respectively. However, we find that peak positions are slightly different, with pyrite shifting about 0.5 eV to lower binding energies. The Fe 2p3/2 and 2p1/2 spin–orbit peaks of marcasite are located at 707.7 eV and 720.5 eV, while these two peaks shift to 707.2 eV and 720.0 eV in pyrite. Previous magnetic susceptibility measurements have shown that both marcasite and pyrite are diamagnetic, and the iron ions in both compounds are in the Fe^2+^ state with a d^6^ low-spin state. The higher Fe binding energy in marcasite may arise from the closer Fe–Fe distances compared to that of pyrite. The high-resolution S 2p XPS spectrum profiles of marcasite and pyrite are nearly the same in shape and peak positions. The two peaks located at around 162.5 and 163.7 eV are assigned to the S 2p3/2 and S 2p1/2 spin–orbit peaks, respectively. The XPS measurement result suggests that the S states in marcasite and pyrite are nearly the same, but that the Fe in marcasite has higher binding energy than that of pyrite.

### 3.4 Isothermal annealing experiments

The transformation kinetics of marcasite to pyrite could so far only be investigated on natural marcasite mineral samples. Detailed studies were performed using an infrared spectroscopic study and *in situ* synchrotron powder X-ray diffraction measurement by Lennie and Yao, respectively.^4,11^ The transformation rate was found to be temperature-dependent and assumed to follow the Arrhenius equation:
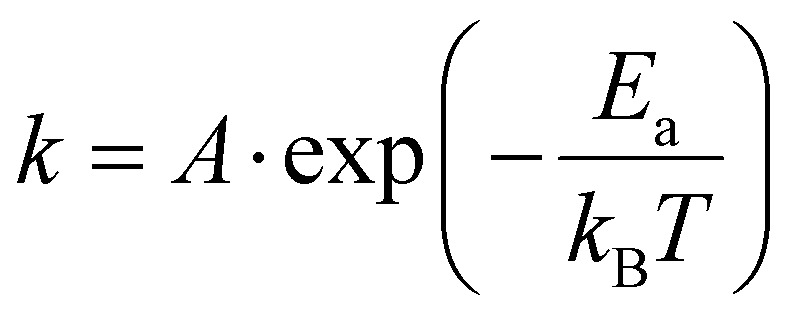
where *A* is an exponential factor, *T* is the absolute temperature, *k*_B_ is the Boltzmann constant, and *E*_a_ is the transition energy barrier. Based on their obtained data, the conversion activation energy from marcasite to pyrite was calculated to be 253(8) kJ mol^−1^ and 380(15) kJ mol^−1^, respectively.^4,11^ The big discrepancy may come from the different measurement techniques or differences in the natural marcasite samples.

At low temperatures, the marcasite to pyrite transformation rate is quite slow, whereas at high temperatures, the complete transformation can occur very fast. Previous investigations showed, at 350 °C, heat treatment of marcasite under vacuum for up to one year did not induce pyrite. In our TG-DTA test, marcasite did not show obvious changes below 340 °C. Therefore, marcasite can be regarded as stable below this temperature in dry conditions.

At 400 °C, we did not observe any trace of pyrite after 300 hours by PXRD measurement, indicating the rate of transformation at this temperature is also very slow. Earlier investigations performed on natural marcasite mineral samples showed marcasite started to transform to pyrite at 416 °C. When annealing marcasite at 430 °C we observed the formation of pyrite by PXRD measurement after 20 hours.

**Fig. 3:**
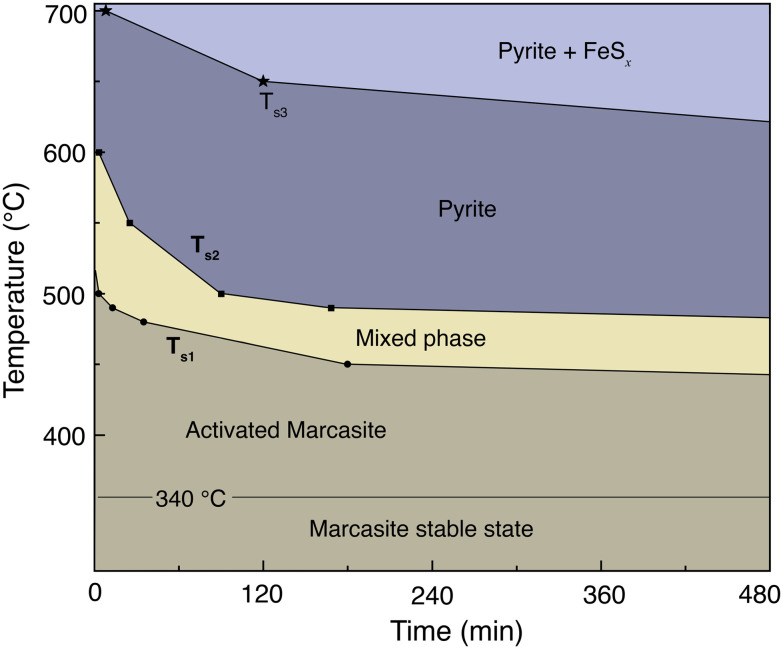
Annealing temperature, time, and phase composition map of marcasite based on our TG-DTA analysis and results of the isothermal annealing experiments at temperatures between 400 and 700 °C within 8 hours.

**Fig. 4:**
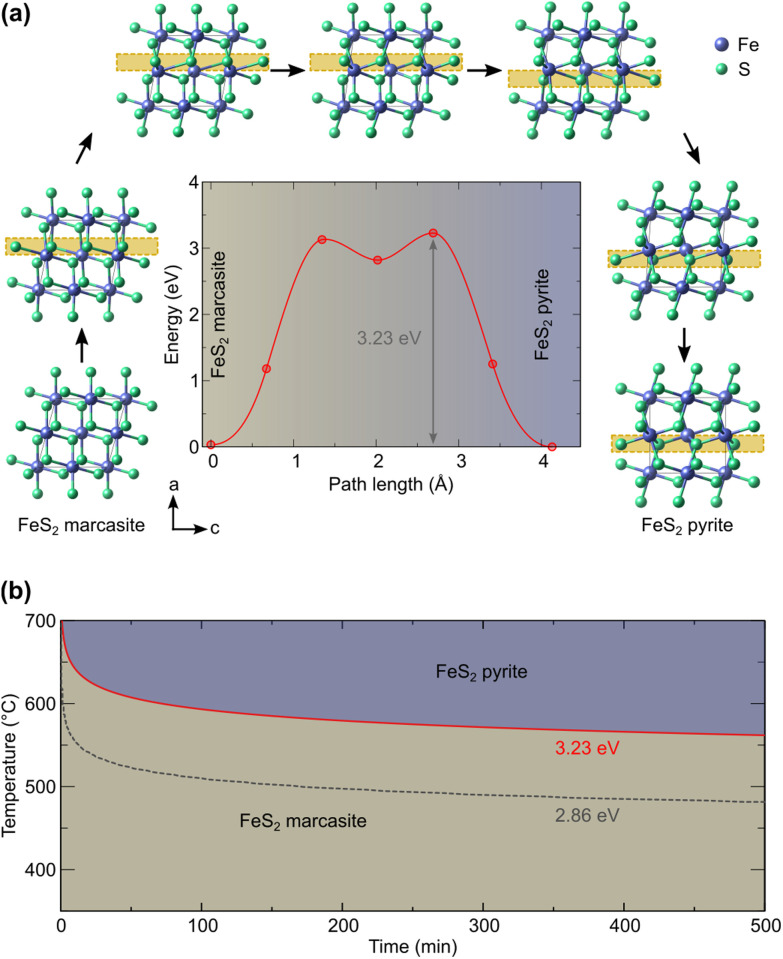
(a) Computed marcasite to pyrite transformation (the main structural changes are highlighted by the yellow box) and energy profile and (b) derived time–temperature diagram.

When the annealing temperature was further increased to 450 °C (S-Fig. 3, ESI†) and 480 °C, we started to observe the formation of pyrite peaks from the PXRD measurements after 170 and 45 minutes, respectively. However, we find that the phase transformation is not completed within 8 hours at these temperatures, the resulting final products being composed of both phases, as shown in Fig. 2(b). At 490 °C, we can observe a complete phase transformation process within 4 hours. As shown in Fig. 2(c), pyrite diffraction peaks start to appear after annealing for 20 minutes, and the transformation was finished after about 170 minutes. At higher temperatures of 500, 550, and 600 °C, the phase transformation started instantly and finished within 80, 30, and 5 minutes, respectively (S- Fig. 4, ESI†). These results indicate that the marcasite to pyrite phase transition is a thermally activated process and that the transition rate increases sharply with superheating temperatures above 430 °C. When the annealing temperature increased to above 650 °C, we observed the co-existence of pyrite and FeS_*x*_ in the NiAs-type structure, consistent with the TG-DTA measurements (S-Fig. 4 and 5, ESI†).

### 3.5 Time–temperature-phase composition transformation map

In Fig. 3, we present an annealing temperature, time, and phase composition map of marcasite based on our TG-DTA analysis and results of the isothermal annealing experiments at temperatures between *T* = 400 and 700 °C within 8 hours. This map is mainly composed of five areas, stable marcasite, activated marcasite, mixed phase, pyrite, and pyrite with FeS_*x*_. Even though marcasite is a thermodynamically metastable phase, its transformation to pyrite is kinetically hindered by the phase transition barrier at temperatures below 340 °C. Therefore, below this temperature, marcasite can be regarded as stable. As the temperature further increases, marcasite starts to absorb more external energy to enter an activated state, where marcasite will convert to pyrite when it gains enough activation energy to overcome the phase transition barrier. In this temperature range, marcasite to pyrite phase transition rate increases sharply with annealing temperature. Below 400 °C, the phase transition is quite slow, which may take many months or even years.

Between 480–550 °C, the phase transition has a moderate transformation rate. We can observe a complete phase transition from marcasite to mixed phase, and finally pyrite within a few hours. At temperatures above 630 °C, pyrite becomes unstable and will decompose to form the NiAs-type FeS_*x*_.

### 3.6 Density functional theory (DFT) calculations

To understand the possible microscopic mechanism and pathways of this polymorphic phase transformation, we conducted SSNEB calculation. An initial SSNEB calculation between pyrite and marcasite yielded a pathway with a second unstable phonon in the transition state structure, corresponding to the bending of the FeS_6_ octahedra. The original transition state is thus not a true saddle point, the eigenvector of the bending mode pointing towards the true saddle point and the real minimum energy pathway. We hence lowered the symmetry of the transition state by freezing in this mode and using this structure as an intermediate image in the SSNEB starting interpolation. The resulting pathway consists of a two-step transformation, with the symmetry-lowered transition state as a local minimum. The first step in this pathway corresponds to a translation of the bottom half of the central octahedral row, whereas the second step applies a similar transformation to the upper half, as shown in Fig. 4(a). The barrier height for this pathway is 3.23 eV, which is 1.21 eV lower than the barrier of the initial NEB without the symmetry-lowered transition state.

Given that in our computational setup, the marcasite phase is 0.03 eV per simulation cell less stable than the pyrite phase, the barrier height for the transformation of marcasite to pyrite has a barrier height of 3.20 eV. To compute the rate constant *k* for a phase transformation with this barrier (Δ*E*_b_), we evaluate the prefactor *via* the computed vibrational partition functions at the ground (*Z*_gs_) and transition state (*Z*_ts_) according to:
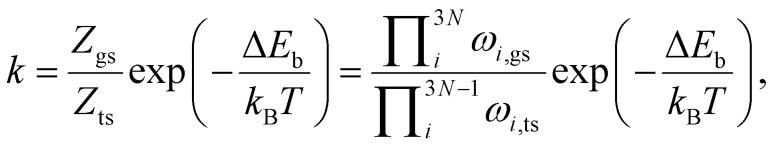
where *k*_B_ is the Boltzmann constant, *T* the temperature, and *ω*_*i*,gs_ and *ω*_*i*,ts_ the phonon frequencies at the ground and transition state respectively. For the transition state, the imaginary mode of the saddle point is excluded. The prefactor amounts to 665.05 THz from our phonon calculations. We then obtain the timescale of the transformation as the inverse of this rate. Evaluating this expression for different temperatures yields the time–temperature diagram in Fig. 4(b).

Our predicted transition temperature in the long-time limit lies around 150 °C higher than the experimentally determined value. A perfect match would be obtained for a barrier of 2.86 eV (shown as the dashed line in Fig. 4(b)). The quantitative disagreement compared to the experiments could stem from the high sensitivity of the predicted time on the barrier, given that the latter appears in the exponential function. Even changes in the order of the DFT error (0.05 eV) shift the predicted transition temperature by more than 10 K. Moreover, our NEB considers the phase transformation to be monolithic, whereas a region-wise transformation (nucleation and growth) could have a lower barrier. Other electronic and quantum effects could also contribute to lowering the barrier, which will be a worthy topic for future research. Nevertheless, our predicted barrier lies within the range of 2.6 eV (ref. 4) to 3.9 eV (ref. 11) determined for natural marcasite samples. Considering this, we deem the predicted pathway and barrier sufficiently reliable to enable, at least, a qualitative comparison with the experiments. Based on our results, it can therefore be ascertained that the transformation of marcasite to pyrite, while thermodynamically favorable, is hindered by a large kinetic barrier of the order of 3 eV and that marcasite can remain stable at room temperature for a very long time.

## 4 Conclusion

In summary, we performed an investigation of the prototypical polymorphic structural transformation from marcasite to pyrite FeS_2_ by combining annealing experiments and theoretical calculations. Based on phase-pure marcasite crystals prepared by a space-separated hydrothermal synthesis technique, we carried out TG-DTA analysis and isothermal annealing experiments. These results allow us to construct an annealing temperature, time, and phase composition map of marcasite, which quantifies the phase transformation kinetics. The theoretical calculations yield a transformation pathway with a barrier of the right order of around 3 eV and show that the transformation from marcasite is kinetically limited at and also above ambient temperatures.

## Author contributions

FvR designed the experiment. KM carried out the experiments and measurements. UA performed the theoretical calculations. KM, UA, and FvR did the data analysis. KM, UA, and FvR wrote the manuscript together.

## Data availability

The data supporting this article have been included as part of the manuscript and the ESI.†

## Conflicts of interest

There are no conflicts to declare.

## Supplementary Material

DT-054-D4DT03447C-s001

DT-054-D4DT03447C-s002

DT-054-D4DT03447C-s003

DT-054-D4DT03447C-s004
