# Supplementary material for: A combined experimental and theoretical study of the prototypical polymorphic transformation from marcasite to pyrite FeS2
Source: Dalton Trans. 2025 Feb 19;54(11):4728–34. doi: 10.1039/d4dt03447c (PMC11837771; doi:10.1039/d4dt03447c)
Supplement: DT-054-D4DT03447C-s003 [file DT-054-D4DT03447C-s003.pdf]

Supplementary Information (SI) for Dalton Transactions.
This journal is © The Royal Society of Chemistry 2025

Supporting Information

A Combined Experimental and Theoretical Study of the Prototypical Polymorphic Transformation from Marcasite to Pyrite FeS_2

KeYuan Ma,† Ulrich Aschauer,‡ and Fabian O. von Rohr∗,¶

†Max Planck Institute for Chemical Physics of Solids, 01187 Dresden, Germany

‡Department of Chemistry and Physics of Materials, University of Salzburg, 5020 Salzburg, Austria

¶Department of Quantum Matter Physics, University of Geneva, CH-1211 Geneva, Switzerland

E-mail: Fabian.vonRohr@unige.ch

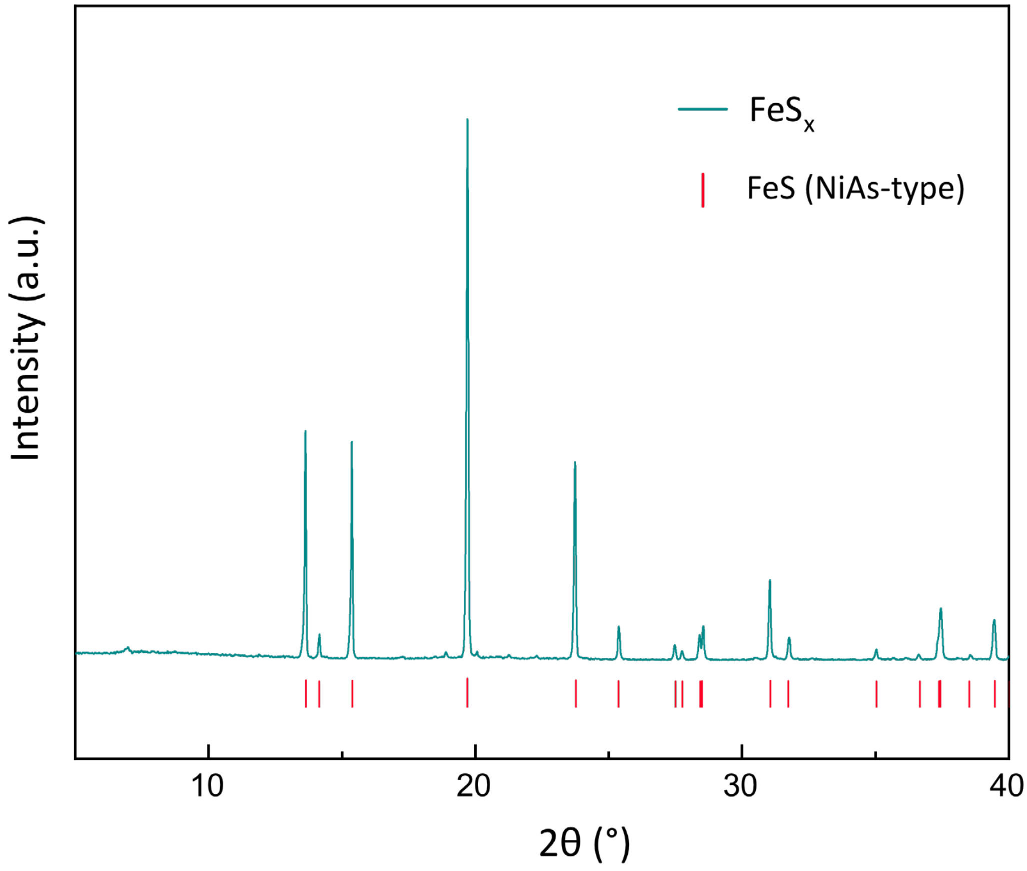

S-Figure 1, PXRD measurement of the residual product of FeS_2 marcasite after the TG-DTA measurement under argon atmosphere.

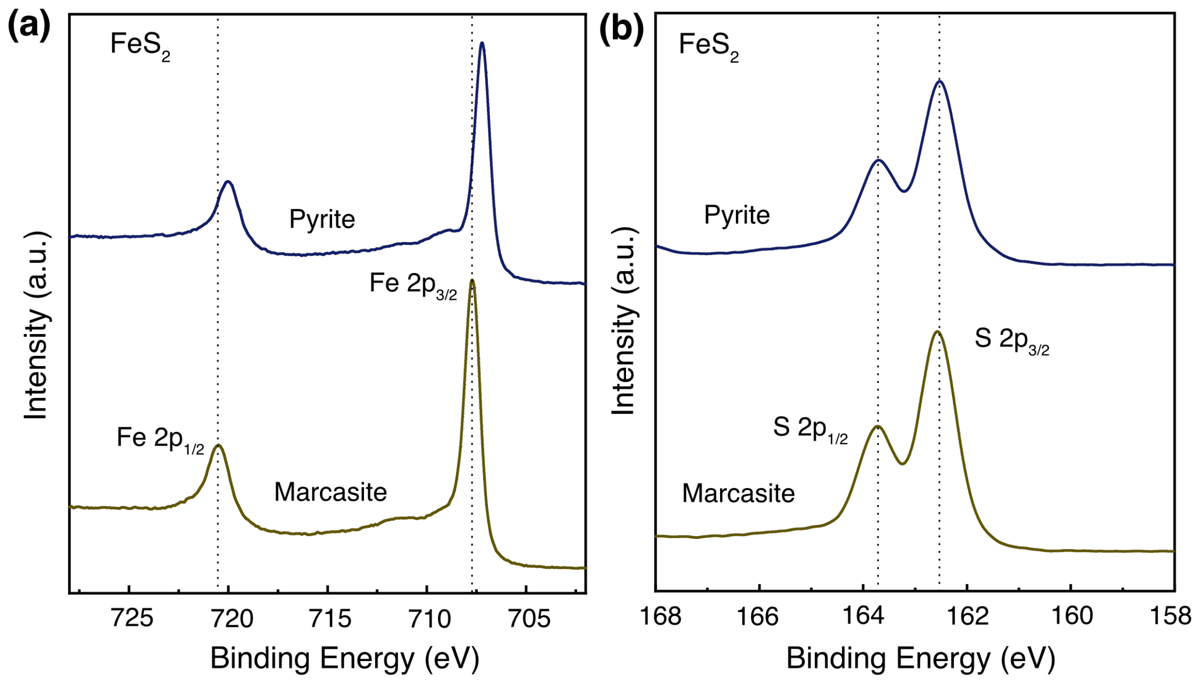

S-Figure 2, X-ray photoelectron spectra show marcasite has higher energy state than pyrite. Comparison of the Fe 2p (a) and S 2p (b) spectra of marcasite and pyrite, respectively.

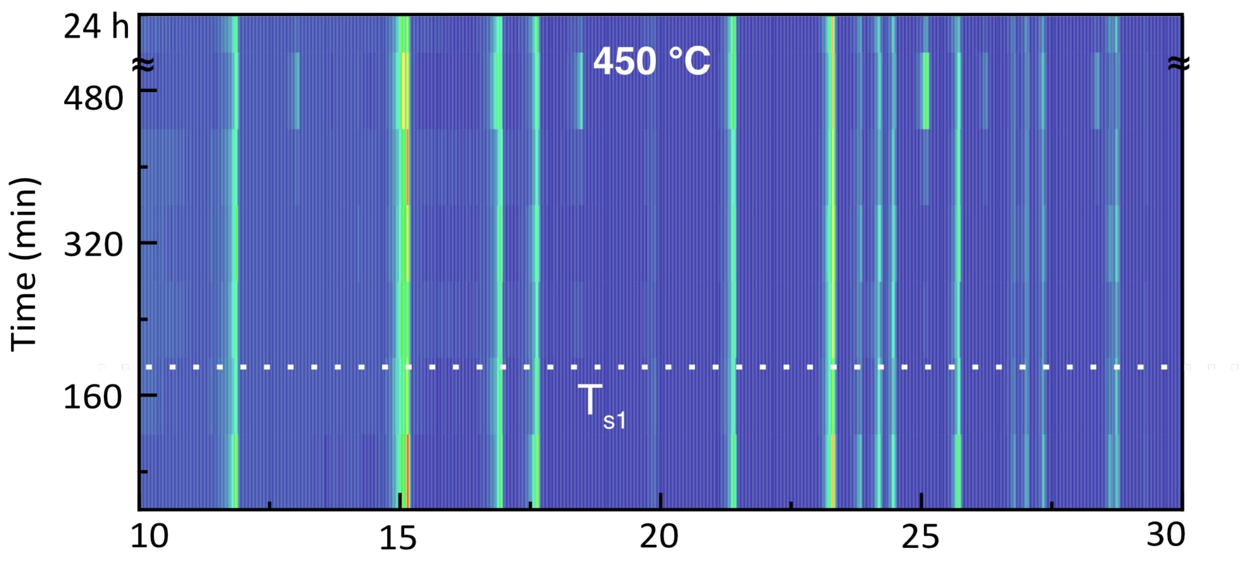

S-Figure 3, Isothermal annealing experiments of lab-prepared marcasite at 450 °C. The T_{s1} line indicates the starting formation time of FeS_2 pyrite.

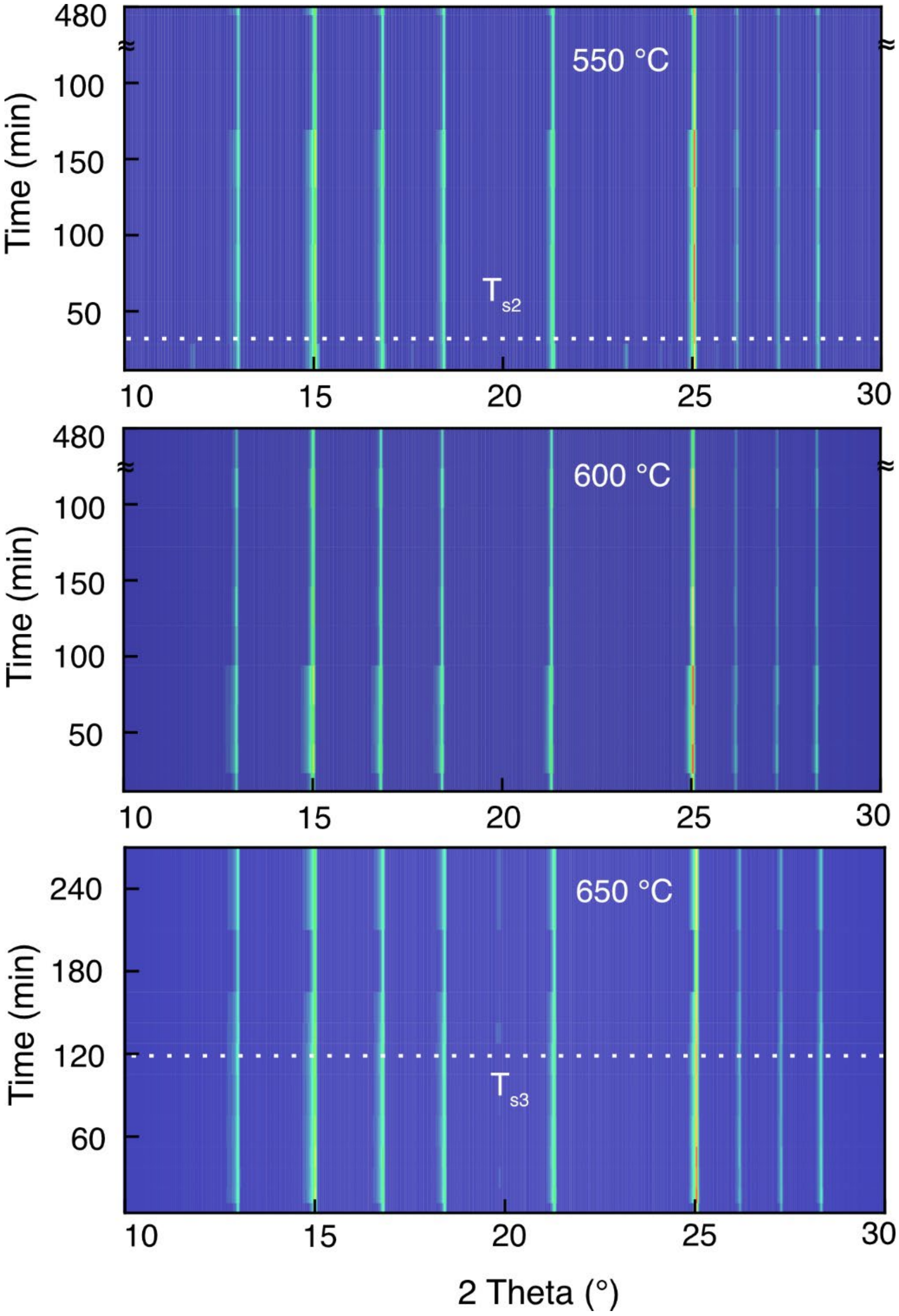

S-Figure 4, Isothermal annealing experiments of lab-prepared marcasite at 550 °C, 600 °C, and 650 °C, respectively.

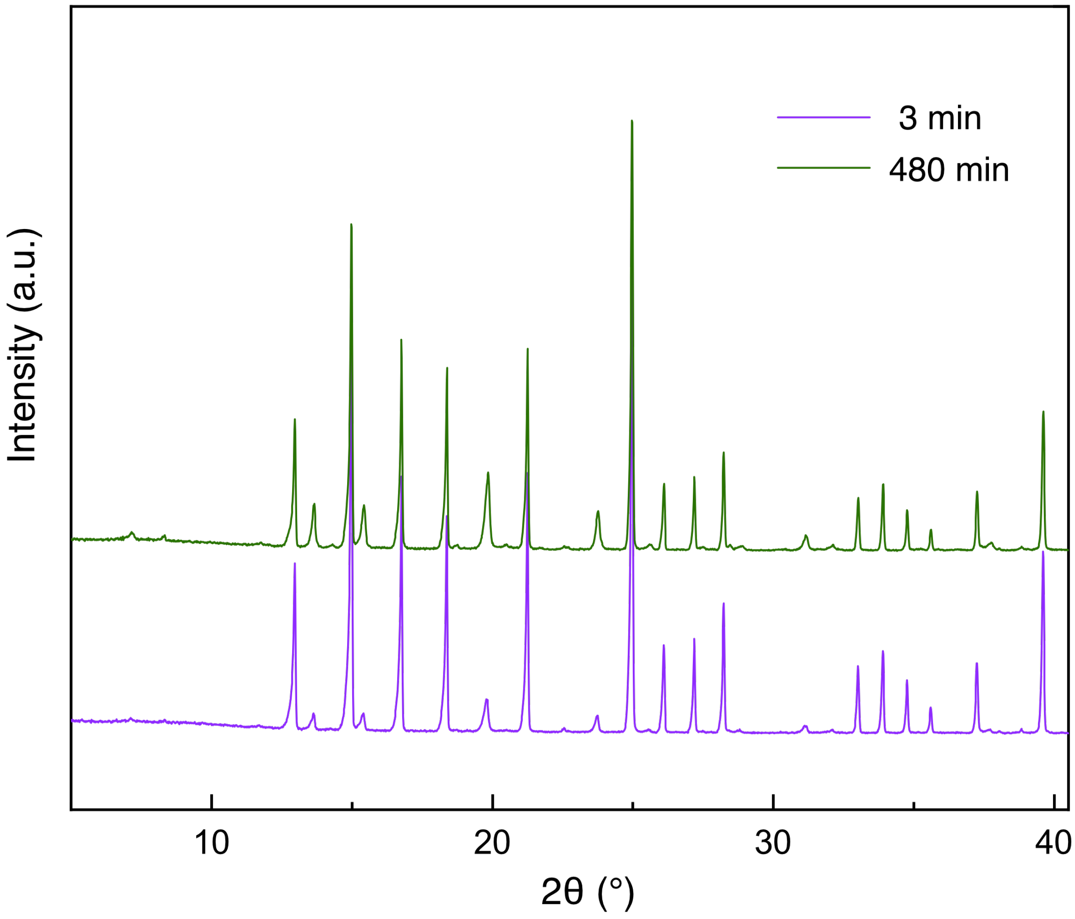

S-Figure 5, PXRD measurements of FeS_2 marcasite after the isothermal annealing experiments at 700°C after 3 and 480 min, respectively. At 700 °C, we observed the co-existence of FeS_2 pyrite and the FeS_x (NiAs-type).

Table 1: Γ point phonon frequencies (in THz) in marcasite, at the two transitions states and in pyrite. The full details including the eigenvectors can be found in the phonopy YAML files in the SI.

Mode	Marcasite	TS 1	TS 2	Pyrite
1	0.00	-5.55	-6.32	0.00
2	0.00	-0.01	-0.01	0.00
3	0.00	-0.01	-0.00	0.00
4	6.27	-0.00	-0.00	6.88
5	6.27	3.46	3.81	6.90
6	6.48	4.68	4.10	6.90
7	7.31	5.47	5.44	6.90
8	7.31	5.89	5.81	7.75
9	8.04	6.45	6.40	7.75
10	9.54	7.38	7.03	9.54
11	9.94	7.69	7.48	9.54
12	9.94	8.39	8.18	9.54
13	9.96	8.44	8.46	10.54
14	10.22	8.84	8.76	10.75
15	10.22	9.06	9.13	10.75
16	10.27	9.38	9.18	10.98
17	10.36	9.53	9.35	10.98
18	10.36	9.75	9.98	10.98
19	10.71	9.93	10.00	11.26
20	10.71	10.21	10.04	11.26
21	11.49	10.27	10.20	11.26
22	11.66	10.64	10.50	11.58
23	11.66	10.77	10.65	11.78
24	11.92	10.77	10.84	11.78
25	11.92	11.02	10.93	11.78
26	12.13	11.45	11.01	12.03
27	12.22	11.63	11.38	12.03
28	12.24	11.77	11.61	12.32
29	12.28	12.06	11.93	12.32
30	12.54	12.13	11.96	12.32
31	12.54	12.59	12.15	12.72
32	12.83	12.97	12.24	12.72
33	13.05	13.23	13.04	12.72
34	13.26	13.42	13.10	13.61
35	13.26	13.74	13.56	13.61
36	14.93	14.58	14.17	13.61
